# Pharmacological management and outcomes of sustained ventricular tachycardia in children and adolescents: a single-center retrospective study

**DOI:** 10.3389/fped.2026.1835079

**Published:** 2026-09-09

**Authors:** Jinghao Li, Hong Wang, Yanlin Xing, Ce Wang, Yunming Xu, Yanqiu Chu

**Affiliations:** Department of Pediatric Cardiology, Shengjing Hospital of China Medical University, Shenyang, China

**Keywords:** antiarrhythmic agents, pediatrics, ventricular tachycardia, left fascicular, cardioversion

## Abstract

**Background:**

Evidence related to the efficacy and prognosis of pharmacologic therapy for sustained ventricular tachycardia (VT) in the pediatric population remains scarce. This single-center retrospective study aimed to evaluate the therapeutic outcomes and drug sensitivity of sustained VT in children and adolescents.

**Methods:**

We analyzed patients hospitalized due to sustained VT at the Department of Pediatric Cardiology, Shengjing Hospital of China Medical University, between February 2018 and September 2025. Patients were prescribed antiarrhythmic drugs according to electrocardiogram (ECG) morphology. Electrocardiographic and echocardiographic data, cardioversion success rate, and adverse events were recorded. Following VT termination, oral antiarrhythmic drugs were initiated for VT prevention.

**Results:**

In total, 29 patients (18 boys, 11 girls) were included, with a mean age of 8.3 ± 4.6 years. The patients were diagnosed with idiopathic VT (*n* = 25), VT associated with myocarditis (*n* = 3), or VT associated with structural heart disease (*n* = 1). According to ECG morphology, patients were classified as presumed left fascicular VT (*n* = 21, 72.4%), outflow tract VT (*n* = 2, 6.9%), polymorphic VT (*n* = 3, 10.3%), and other-origin VT (*n* = 3, 10.3%). A total of 38 sustained VT episodes occurred among these patients. Four episodes of VT terminated without antiarrhythmic intervention, while the remaining 34 episodes were successfully cardioverted under medical intervention, with monotherapy effective in 25 episodes (73.5%). Intravenous verapamil was effective for presumed left fascicular VT (success rate of 92.3%). During a mean follow-up period of 2.1 ± 1.7 years, 41.4% of the patients had complete resolution of VT with drug discontinued, while the remainders still required antiarrhythmic drugs. Drug therapy alone resulted in a higher complete resolution rate in patients with infantile onset (<1 year) compared to those with older onset (66.6% vs. 13.0%, *P* = 0.006). Transient, reversible cardiac dysfunction occurred in only three patients. All patients maintained cardiac function well during follow-up, and no sudden cardiac death was recorded.

**Conclusion:**

Idiopathic VT is the predominant type of sustained VT in children and adolescents. In this study, the vast majority of the cases were left fascicular VT. Patients with presumed left fascicular VT exhibit sensitivity to intravenous verapamil, although clinicians must remain vigilant of transient hypotension. The long-term follow-up results revealed an overall favorable prognosis in pediatric VT.

## 1 Introduction

Although ventricular tachycardia (VT) is uncommon in the pediatric population, it is potentially life-threatening. A survey based on school-aged children in Japan reported an incidence of VT ranging from 0.2 to 0.8 per 10,000 individuals (1), while a single-center retrospective study from the US documented an incidence of 1.1 per 100,000 individuals (2). Due to the abnormal hemodynamics caused by VT, sudden cardiac death and cardiac dysfunction can occur. Evidence indicates that the incidence of arrhythmia-induced cardiomyopathy caused by ventricular arrhythmia is 6.8% (3, 4). Currently, studies on pharmacological management of pediatric VT are scarce. The purpose of this research was to summarize the treatment strategies and long-term follow-up outcomes in pediatric VT. Additionally, we sought to determine the clinical characteristics of VT in this population and evaluate the efficacy and safety of different antiarrhythmic drugs.

## 2 Materials and methods

### 2.1 Study subjects

This single-center, retrospective study enrolled children who were hospitalized with sustained VT at the Department of Pediatric Cardiology, Shengjing Hospital of China Medical University, between February 2018 and September 2025.

The inclusion criteria were as follows: (1) age ≤14 years; (2) sustained VT secondary to various etiologies requiring medical intervention.

The exclusion criteria were as follows: (1) patients with an electrocardiogram (ECG) exhibiting idioventricular escape rhythm only; (2) non-sustained VT only.

### 2.2 Definition of ventricular tachycardia (1, 5)

- Sustained VT was defined as VT lasting ≥30 s or requiring urgent termination due to hemodynamic compromise.
- VT origin localization was determined according to ECG morphology during VT as follows:
  ○ Right ventricular (RV) origin: VT presenting with a left bundle branch block (LBBB) morphology in lead V1.
  ○ Left ventricular (LV) origin: VT presenting with a right bundle branch block (RBBB) morphology in lead V1.
  ○ Presumed outflow tract VT (OT-VT): Characterized as a wide QRS complex with LBBB morphology; high amplitude of the R wave in leads II, III, and aVF; and the precordial transition occurring later than leads V2–3.
  ○ Presumed left fascicular VT: Typically exhibiting a relatively narrow QRS duration. A RBBB morphology with left axis deviation (left anterior fascicular block pattern) suggests a left posterior fascicular origin, whereas a RBBB morphology with right axis deviation (left posterior fascicular block pattern) suggests a left anterior fascicular origin.
  ○ Polymorphic VT: Characterized by the presence of ≥2 different QRS morphologies during VT.

### 2.3 Baseline clinical data

Demographic and clinical data were collected, including age, height, weight, age at VT onset, frequency of VT episodes, causation factors, duration of VT episodes, symptoms, medical history, and any concomitant underlying diseases.

### 2.4 Examinations

- ECG Routine examination of 12-lead electrocardiogram was performed at resting status (paper speed, 25 mm/s; voltage, 10 mm/mV). PR interval, QRS duration, QTc (QT duration corrected by RR interval) were recorded.
- Two-dimensional echocardiography: Evaluated cardiac structure and function, including left ventricular end-diastolic diameter and left ventricular systolic function.
- 24-h Holter monitoring: Assessed overall heart rhythm and heart rate characteristics.
- Cardiac magnetic resonance imaging (CMR): Both T1 and T2 mapping and contrast-enhanced scans were performed to evaluate the presence of myocardial edema, perfusion defects, and myocardial fibrosis/scarring.
- Exercise stress testing (EST): In children (≥6 years old) capable of cooperating, a treadmill exercise test was performed according to the Bruce protocol.

### 2.5 Treatment protocols (6–8)

- Acute termination of sustained VT: Pharmacological therapy was adopted based on the ECG morphology during VT. The classification of drugs was according to Vaughan Williams method.
  Presumed left fascicular VT: Intravenous verapamil (0.1 mg/kg intravenous push slowly) was selected as the first-line agent. In this study, intravenous or oral verapamil was only used in children older than 1 year-old. For infants with presumed left fascicular VT, other antiarrhythmic drugs were adopted instead of verapamil.
  Other-origin VT: Intravenous propafenone (loading dose of 1 mg/kg and maintenance dose of 7 µg/kg/min) or amiodarone (loading dose of 5 mg/kg and maintenance dose of 10 mg/kg/day).
  Unstable hemodynamic VT: Electrical cardioversion was adopted.
- Prophylactic therapy: Following VT termination, oral antiarrhythmic drugs were initiated for VT prevention as follows:
  Propafenone: 15 mg/kg/day, divided three times daily (Q8h).
  Verapamil: 4−8 mg/kg/day, divided three times daily (Q8h).
  Sotalol: 5.5 mg/kg/day, divided twice daily (Q12h).
  Metoprolol tartrate: 1 mg/kg/day, divided twice daily (Q12h).
  Propranolol: 1 mg/kg/day, divided three times daily (Tid).
- Outcomes evaluation: The efficacy of the medication was assessed. Safety was evaluated, including adverse events, atrioventricular conduction block, intraventricular conduction delay, and corrected QT interval prolongation.

### 2.6 Follow-up

The patients were scheduled for follow-up at 1, 3, 6, and 12 months after discharge and subsequently every 6 months. At each visit, clinical symptoms, VT recurrence, body weight, and drug dosage were recorded. A standard 12-leads ECG, echocardiography, and 24-h Holter monitoring were accomplished during the clinical investigation.

For the patients who had no VT recurrence under treatment longer than 6 months (no symptoms related to VT or VT could not be proven by 24-hour Holter monitoring), the antiarrhythmic drug dosage was reduced gradually until discontinued. The drug tapering principle was as follows: (1) the number of antiarrhythmic drugs was decreased firstly; (2) the drug dosage was then reduced by half until discontinued completely; and (3) if there was no recurrence of VT after 3 months, drug tapering continued.

“VT completely controlled” was defined as no symptoms or VT could not be proven clinically under regular antiarrhythmic treatment. “VT completely resolved” was defined as no symptoms or VT could not be proven clinically 6 months after drug therapy discontinued. “VT partially controlled” was defined as the frequency of symptoms related to VT or the sustained VT episodes decreased but still recurred (more than once within 3 months) regardless of antiarrhythmic treatment. “Drug refractory” was defined as recurrent sustained VT episodes (more than once per month) regardless of multiple antiarrhythmic drugs used.

### 2.7 Statistical analysis

Statistical analyses were performed using SPSS (version 22.0). Continuous variables with a normal distribution are presented as mean ± standard deviation and those with a non-normal distribution are presented as median (interquartile range, IQR), while categorical variables are presented as number (percentage). We used the chi-square test for categorical variables comparison between two groups. The risk factors were examined using binary regression analysis. Due to an insufficient sample size and number of outcome events, multivariable modeling was not feasible; thus, only univariable modeling was used. The comparison of variables pre-and post-medication was performed using a paired *t*-test. A two-tailed *P*-value < 0.05 was considered statistically significant.

## 3 Results

### 3.1 General

A total of 29 patients (18 boys, 11 girls) were enrolled. The mean age was 8.3 ± 4.6 years (range: 1 month to 13 years). All the patients were hospitalized due to sustained VT. Patients were diagnosed with idiopathic VT (*n* = 25, 86.2%), VT associated with myocarditis (*n* = 3, 10.3%), and VT associated with structural heart disease (*n* = 1, 3.4%).

Among these patients, 25 (86.2%) experienced symptoms during VT episodes, including palpitations, chest tightness, chest pain, and dizziness. Syncope occurred in one patient. Four patients (13.8%) were asymptomatic. VT was induced by vigorous exercise or physical exertion (*n* = 11) and fever (*n* = 7). One patient presented with sustained VT caused by poor glucose control due to diabetes.

### 3.2 Clinical examinations

#### 3.2.1 ECG

**Figure 1:**
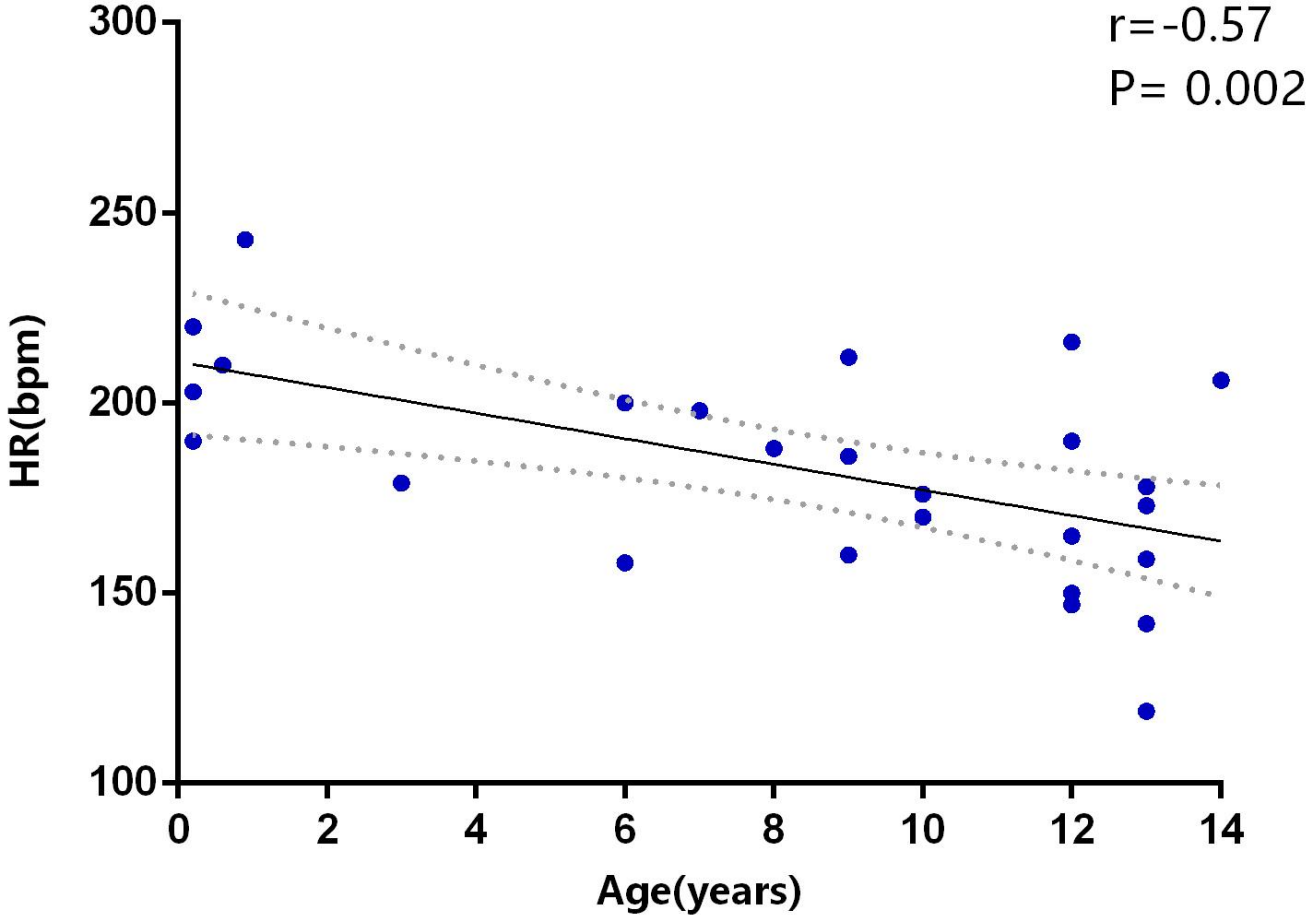
Relationship between heart rate during VT and patient's age.

For the patients who experienced multiple VT episodes, the same ECG morphology was consistently exhibited. The ECG morphology of VT was LBBB in 3 patients, RBBB in 24 patients, and unclassifiable polymorphic in 2 patients. The mean QRS duration during VT was 123 ± 19 ms, with a mean ventricular rate of 182 ± 27 beats per minute. The ventricular rate during VT was related negatively to age (Figure 1). Based on the ECG morphology, the VT cases were classified as presumed left fascicular VT (*n* = 21, 72.4%), presumed outflow tract VT (OT-VT, *n* = 2, 6.9%), polymorphic VT (*n* = 3, 10.3%), or VT of other origin (*n* = 3, 10.3%).

#### 3.2.2 24-h Holter monitoring

Following VT termination, all patients underwent 24-h Holter monitoring. Occasional premature ventricular complexes (PVCs, total <100/day) were found in four patients, frequent PVCs were found in two patients, and transient non-sustained VT was found in three patients.

#### 3.2.3 Exercise stress testing

Exercise stress testing was conducted in nine patients. VT (presumed left fascicular VT) was induced in only one patient.

#### 3.2.4 Echocardiography

Initial echocardiography revealed the following abnormalities in six patients (20.7%):

- Three patients were diagnosed with left ventricular dilation and cardiac insufficiency secondary to sustained VT. The abnormality improved following VT termination.
- A small size of atrial septal defect (diameter as 3 mm) was detected in one patient with normal-sized of cardiac chambers and normal cardiac function.
- Increased myocardial echogenicity was detected in one patient with myocarditis.
- Congenital mitral valve abnormality was detected in one patient with normal cardiac function.

#### 3.2.5 Cardiac magnetic resonance imaging

The CMR examination was conducted in 18 patients. The following abnormalities were detected in three patients with myocarditis:

- A slightly increased T2 signal in the region of the LV lateral wall was detected in one patient with polymorphic VT.
- Late gadolinium enhancement in the infer-septal LV wall region was detected in one patient with LV-origin VT.
- Abnormal myocardial enhancement was detected in one patient with OT-VT.

### 3.3 Treatment and outcomes

**Figure 2:**
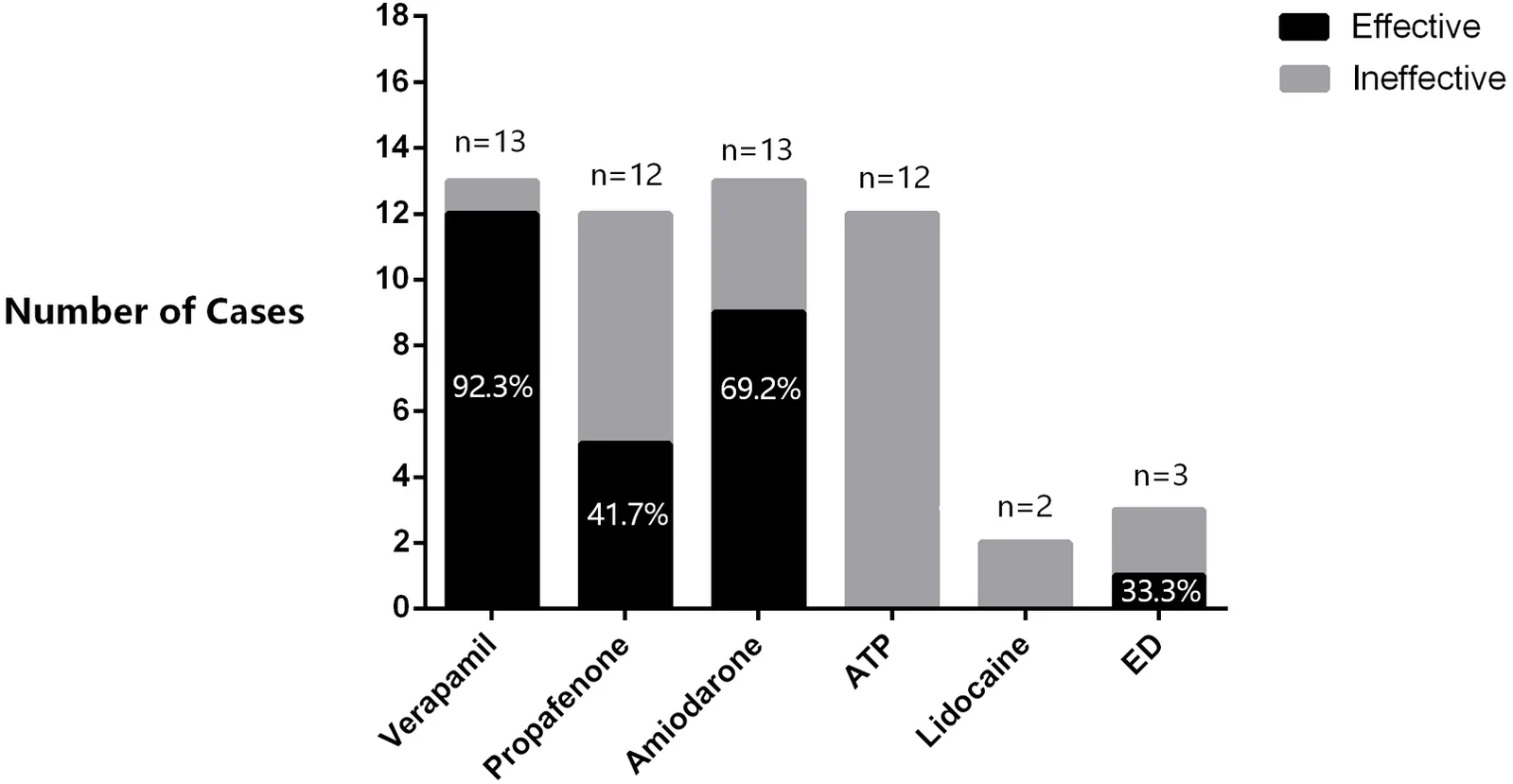
Cardioversion success rates among different antiarrhythmic agents. ATP, adenosine triphosphatase; ED, electrical cardioversion.

**Figure 3:**
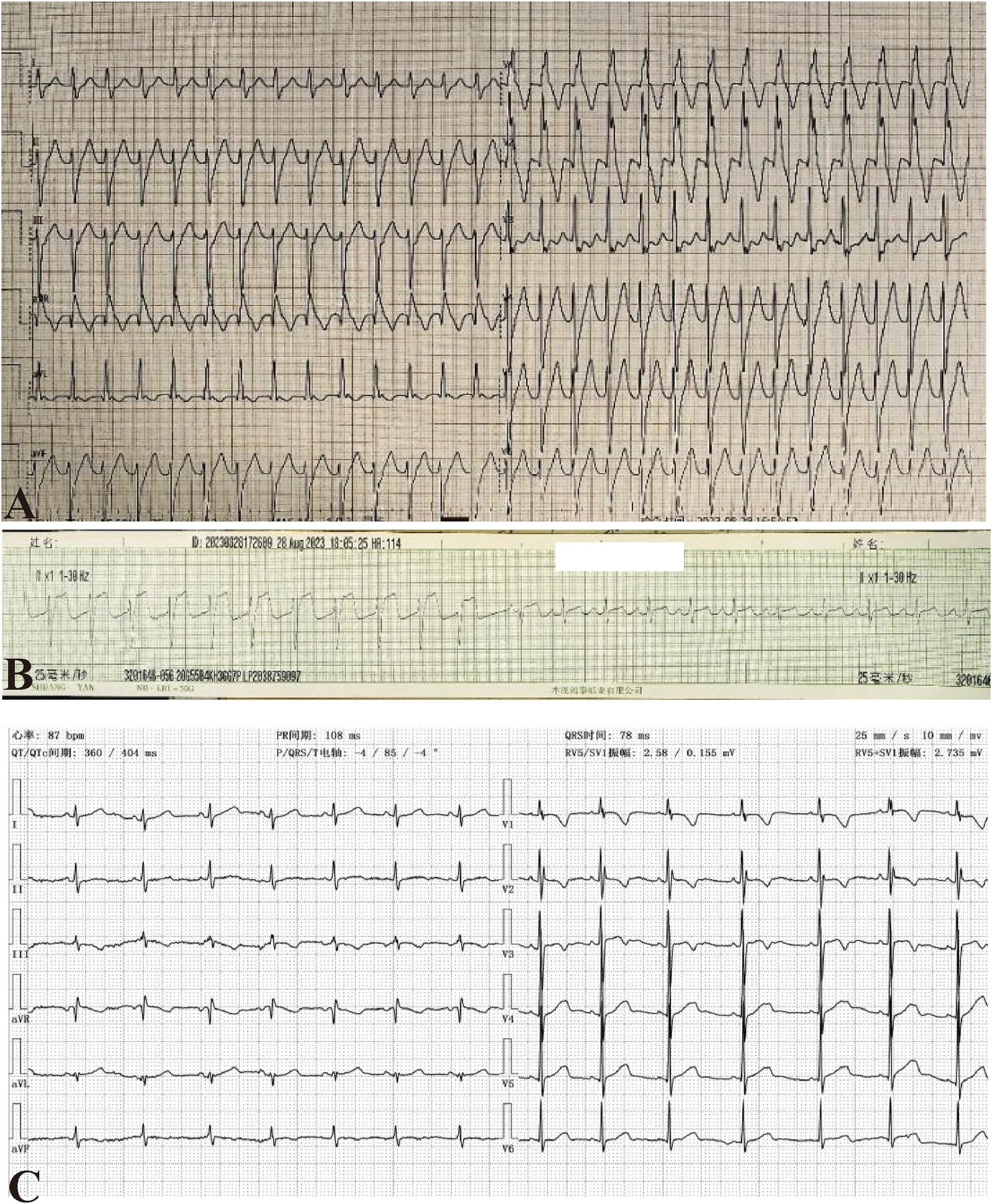
ECGs during cardioversion in a 6-year-old girl with presumed left fascicular VT. **(A)** ECG during VT, a right bundle block pattern in lead V1 and a left anterior fascicular block pattern, with the QRS duration of 114 ms and ventricular rate of 200bpm. **(B)** Successful cardioversion occurred during verapamil infusion. **(C)** ECG following VT termination showing sinus rhythm with a heart rate of 87bpm and a T wave depression.

**Figure 4:**
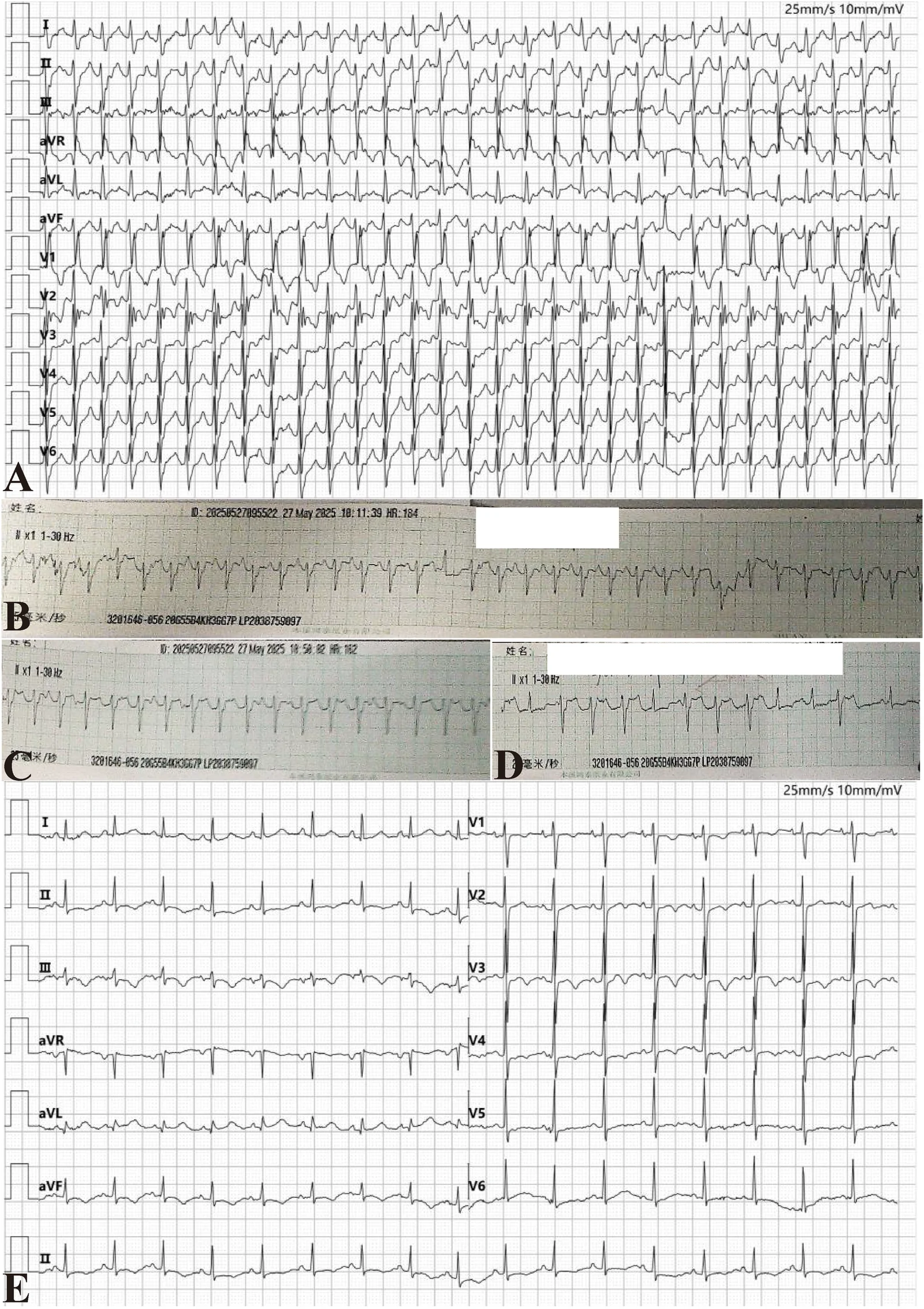
ECGs during cardioversion in an 8-year-old boy with presumed left fascicular VT. **(A)** ECG during VT, in which the phenomena of ventricular-atrial separation and ventricular capture can be observed. There is a right bundle block pattern in lead V1 and a left anterior fascicular block pattern, with a QRS duration of 118 ms and ventricular rate of 188bpm. **(B)** ATP infusion is shown to be ineffective, with no change in the ECG. **(C)** and **(D)** ECGs in the initial period and during verapamil infusion, respectively. The frequency of VT decreases and sinus rhythm increases gradually. **(E)** ECG following VT termination, showing sinus rhythm with a heart rate of 103bpm and T wave depression.

**Figure 5:**
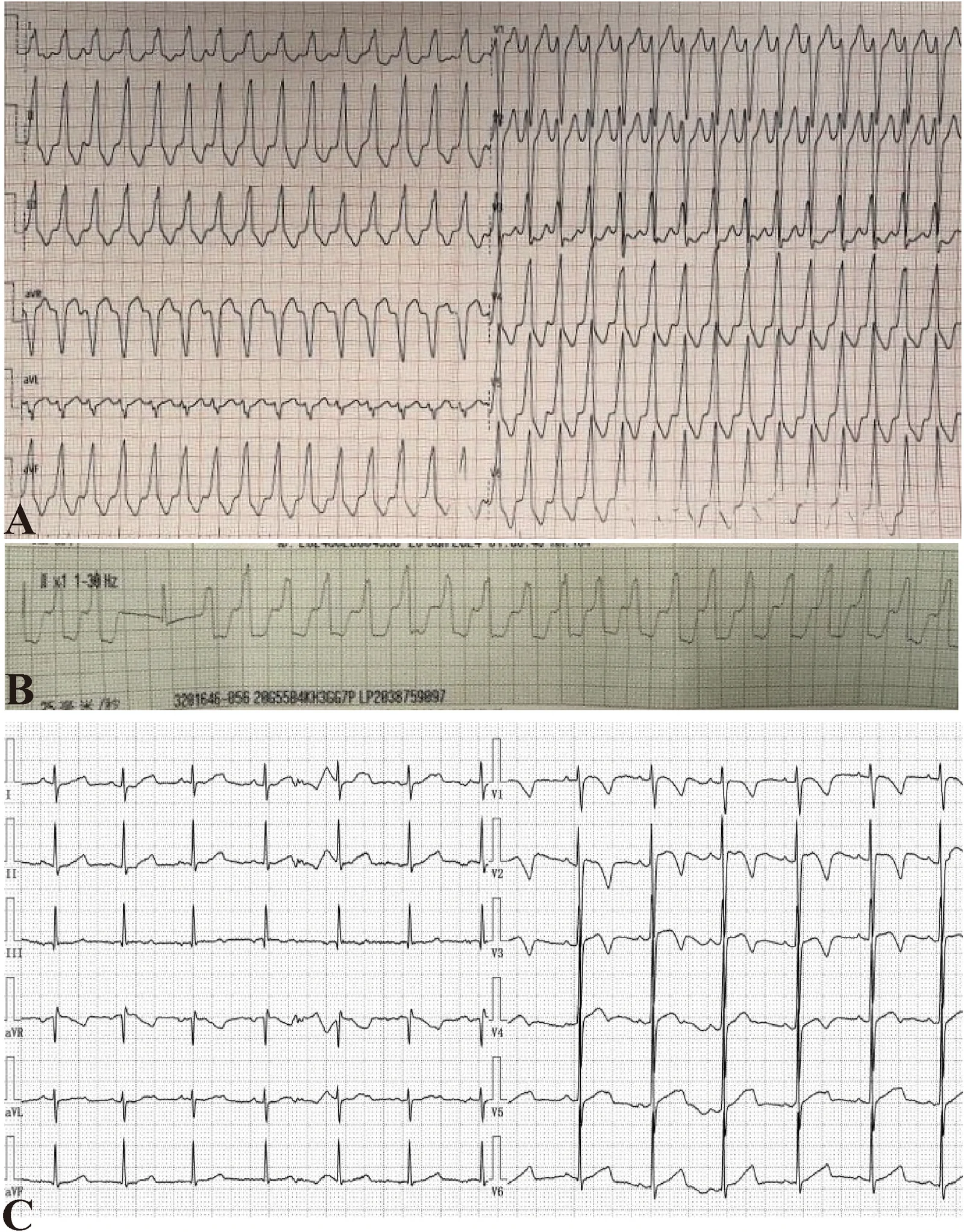
ECGs during cardioversion in a 6-year-old boy with presumed outflow tract VT. **(A)** ECG during VT showing a light bundle block pattern in lead V1; high R wave amplitude in leads II, III, and aVF; negative QRS vector direction in lead aVL; and the precordial transition at lead V3, with a QRS duration of 120 ms and ventricular rate of 200 bpm. **(B)** Ineffective cardioversion during ATP infusion. **(C)** ECG shows sinus rhythm after cardioversion by amiodarone.

A total of 38 sustained VT episodes occurred among all the patients. Four episodes of VT terminated without antiarrhythmic intervention, while antiarrhythmic therapy was administered for the remaining episodes. Monotherapy achieved successful cardioversion in 25 episodes (73.5%), while combination therapy (≥2 kinds of drugs) achieved successful cardioversion in the remaining episodes (26.5%). The cardioversion success rates of the different antiarrhythmic agents were shown in Figure 2 and listed as follows:

- Verapamil: Intravenous verapamil was administered in a total of 13 VT episodes (among eight patients with presumed left fascicular VT). Successful cardioversion occurred during verapamil infusion in 12 VT episodes. The median time for cardioversion was 8 min (IQR: 7, 10 min). Representative cases were shown in Figures 3, 4, respectively. Transient hypotension occurred in five episodes (38.5%), and all resolved immediately following the drug administered.
- Amiodarone: Intravenous amiodarone was administered in a total of 13 VT episodes (nine patients). Successful cardioversion occurred in nine episodes when using amiodarone alone. Cardioversion occurred only once during the loading dose infusion; however, cardioversion occurred during the maintenance dose infusion in the other cases. The median time for cardioversion was 5.5 h (IQR: 2, 17 h). A representative case was shown in Figure 5. One patient developed cardiogenic shock and was cardioverted successfully during cardiopulmonary resuscitation. Amiodarone combined with other antiarrhythmic drugs was administered in three episodes.
- Propafenone: Intravenous propafenone was administered in a total of 12 VT episodes (among seven patients). In five episodes, successful cardioversion occurred during the loading dose infusion. The median time for cardioversion was 10 min (IQR: 10, 10 min). One patient (8.3%) experienced transient hypotension during the propafenone infusion.
- Other agents: Adenosine (*n* = 12 episodes) and lidocaine (*n* = 2 episodes) were ineffective.
- Electrical cardioversion: Electrical cardioversion was attempted in three episodes, with success in one (polymorphic VT) and unsuccess in two (presumed left fascicular VT and polymorphic VT, respectively).

In summary, verapamil achieved a 92.3% success rate of cardioversion in presumed left fascicular VT. In presumed OT-VT (two episodes), intravenous propafenone and amiodarone achieved successful cardioversion respectively. In polymorphic VT (three episodes), electrical cardioversion was successful in one episode, and intravenous amiodarone was successful in two.

### 3.4 Follow-up

The mean follow-up duration was 2.1 ± 1.7 years (range: 2 months to 7.5 years), with 75.9% longer than 1 year. One patient was lost to follow-up. After discharge, oral antiarrhythmic drugs were prescribed in 27 patients. Among these patients, VT was completely controlled in 16 (59.3%), partially controlled in 7 (25.9%), and drug refractory in 4 (14.8%). Monotherapy was prescribed in 19 patients (70.4%), including verapamil (*n* = 15), amiodarone (*n* = 1), or propafenone (*n* = 3). Combination therapy (≥2 kinds of antiarrhythmic drugs) was prescribed in eight patients (29.6%).

Six patients (20.7%) underwent radiofrequency ablation due to frequent VT recurrence. Two patients (left posterior fascicular VT and RV- origin VT, respectively) required a second procedure due to VT recurrence after the primary procedure and their VT was resolved finally. One patient with left posterior papillary muscle origin VT experienced VT recurrence after the primary procedure and had to continue with oral antiarrhythmic drug therapy. In the remaining patients (all with left posterior fascicular VT), the VT was resolved after the primary procedure.

At last follow-up, VT had completely resolved in 12 patients (41.4%). The median time of drug discontinuation was 6 months (IQR: 2.5–15 months) among these patients. The remaining patients still require antiarrhythmic treatment. Combination therapy was required in only one patient, while monotherapy was required in the others. All the patients maintained good cardiac function, and no death was recorded. During the follow-up, three adverse events were documented in the patients with oral verapamil, including prolonged PR interval (*n* = 1), sinus arrest (*n* = 1), and hypotension (*n* = 1). They recovered following drug dosage reduced or discontinued. No adverse drug reactions were recorded in the other patients. There were no differences in the mean PR interval, QRS duration, or QTc values between baseline and follow-up among the patients with antiarrhythmic drugs (Table 1).

**Table 1:** Comparison of PR interval, QRS duration and QTc values at baseline and follow-up.

Variate	Baseline (pre-oral AAD)	Follow-up (oral AAD)	*P*-value
PR interval (ms)	138 ± 21	141 ± 18	0.533
QRS duration (ms)	86 ± 12	87 ± 8	0.451
QTc (ms)	406 ± 25	402 ± 26	0.544

AAD, antiarrhythmic drug.

Six patients (20.7%) had VT onset during infancy (<1 year of age). Antiarrhythmic drugs were prescribed and none of these patients underwent radiofrequency ablation. VT was completely resolved in four of these six patients during follow-up. The complete VT resolution rate due to antiarrhythmic drug therapy (despite radiofrequency ablation) was higher in the infantile-onset group compared to that of the older-onset group (66.6% vs. 13.0%, *P* = 0.006).

**Figure 6:**
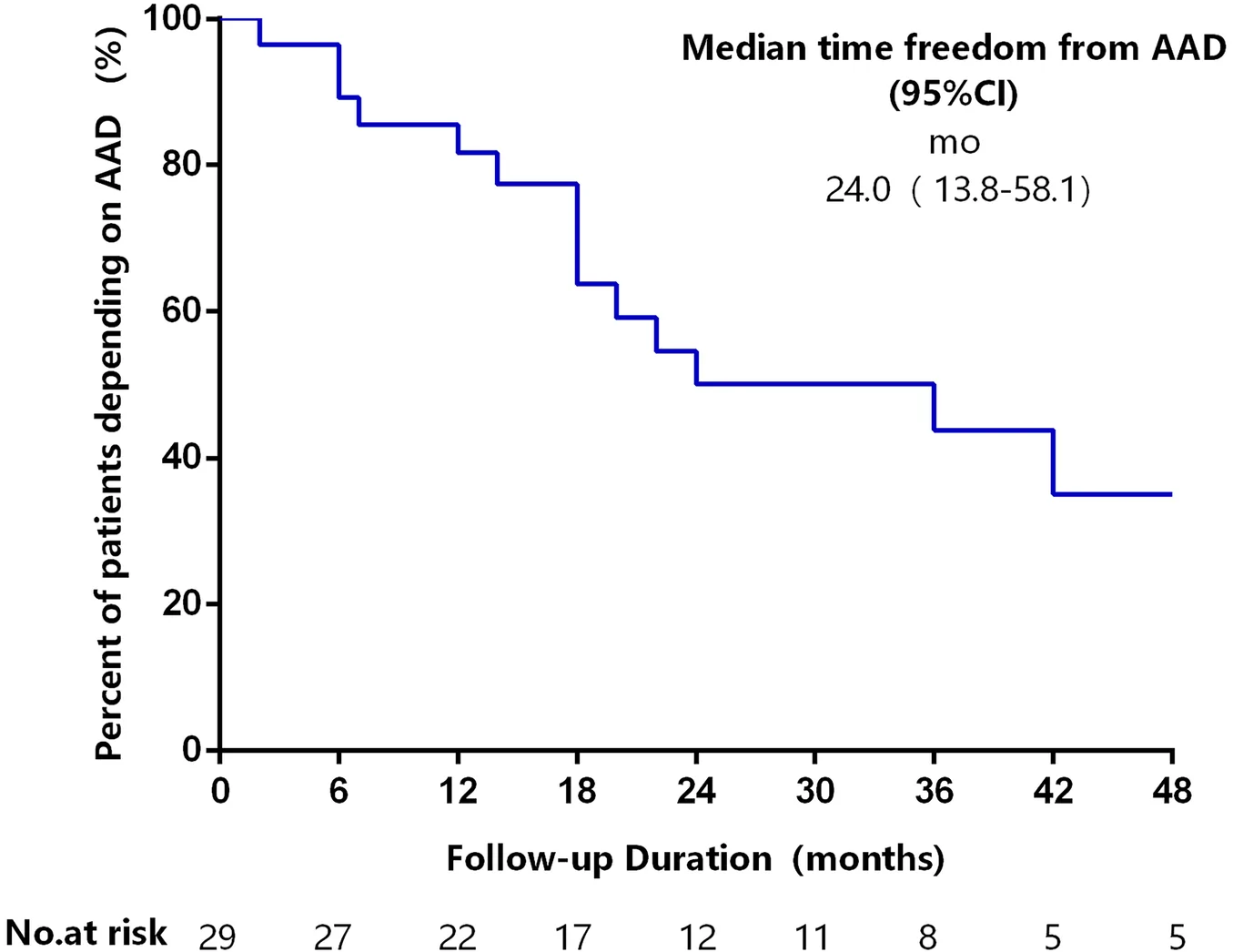
The probability of freedom from antiarrhythmic drug to prevent VT recurrence. AAD, antiarrhythmic drugs.

The factors influencing VT recurrence were studied. Onset as school age and ventricular rate during VT were significantly associated with VT recurrence during follow-up (Table 2). The probability of freedom from antiarrhythmic drug to prevent VT recurrence was illstrated as Kaplan–Meier curve (Figure 6).

**Table 2:** Influencing factors associated with VT recurrence regardless of treatment.

Variate	Univariate analysis	
	OR (95% CI)	*P*-value
School-aged	10.00 (1.02–97.50)	0.048
Gender	0.54 (0.11–2.67)	0.455
Presence of symptoms	3.33 (0.31–34.83)	0.315
ECG morphology	1.62 (0.08–29.78)	0.744
QRS duration during VT	1.03 (0.98–1.08)	0.205
Ventricular rate during VT	0.94 (0.89–0.98)	0.015
VT triggered by excitation of SNS	0.79 (0.15–4.02)	0.782

SNS, sympathetic nervous system.

## 4 Discussion

Idiopathic VT is the most common type of pediatric VT (9). However, structural cardiac abnormalities, cardiomyopathies (e.g., dilation, hypertrophy, or ischemia), and myocarditis could cause VT (10–17). In our study, we classified 3 patients with LBBB morphology and 24 patients with RBBB morphology according to their ECGs during VT. Thus, LV-origin VT make up the majority, with idiopathic left fascicular VT accounting for 72.4%. One patient was diagnosed with presumed left anterior fascicular VT, while the others were all diagnosed with left posterior fascicle VT. Owing to the similar ECG morphology, it is difficult to distinguish between fascicular and papillary muscle-origin VT. Without intracardiac electrophysiological mapping, precise differentiation between left fascicular and papillary muscle-origin VT was not feasible in this study. Thus, we defined this subtype as presumed left fascicular VT.

In the results, VT was triggered by excitation of the sympathetic nervous system (e.g., vigorous exercise or fever) in 62.1% of the patients. Studies have reported that the sensitivity of exercise stress testing for inducing ventricular arrhythmias ranges from 32% to 65% (18–21). In our study, a positive result occurred in one of nine patients. For this patient, non-sustained VT (presumed left fascicular VT) was induced during the testing.

In our study, 86.2% of patients experienced symptoms, including chest tightness, pain, and palpitations. One infant who was diagnosed with idiopathic polymorphic VT presented with syncope. Studies had indicated that patients with VT associated with syncope tend to have a significantly faster ventricular rate compared to those without syncope (22). Furthermore, patients with underlying cardiomyopathy or channelopathies who experience syncope usually have a high risk of sudden cardiac death (24%∼35%). However, idiopathic VT rarely leads to sudden cardiac death (9, 16, 22). Evidence suggests that LV-origin VT is more likely to cause symptoms than RV-origin VT (67% vs. 25%) (23). Our results indicated that sustained VT in children and adolescents frequently causes tachycardia-related symptoms, whatever LV or RV origin. Furthermore, VT should be especially suspected in children who experienced unexplained syncope.

Patients with sustained VT or frequent non-sustained VT have the potential to develop tachycardia-induced cardiomyopathy (4). In our study, three patients developed LV dilation and systolic dysfunction due to long time of sustained VT. These echocardiographic abnormalities were resolved following VT termination. This indicates that VT should be terminated as early as possible to prevent irreversible cardiac dysfunction.

The primary mechanisms of tachycardia include automaticity, reentry, and triggered activity. Based on ECG features and pharmacological response, VT would be classified as follows: adenosine-sensitive VT, verapamil-sensitive VT, and propranolol-sensitive VT (7). In our cohort, 72.4% of patients were diagnosed with idiopathic left fascicular VT (also named verapamil-sensitive VT). The mechanism is related to micro- reentry circuit, which is located between the Purkinje network and the slow conduction zone of the LV myocardium. It is illustrated by the presence of Purkinje potential and a late diastolic potential during intracardiac electrophysiological mapping. Idiopathic left fascicular VT is highly sensitive to verapamil but can be resistant to adenosine or vagal maneuvers. The European Heart Rhythm Association (EHRA) guideline recommends that intravenous verapamil was recommended for children with left fascicular VT (8). A multicenter study found that the efficacy rate was 80% which used intravenous calcium antagonists for left fascicular VT termination (24). In our cohort, intravenous adenosine was ineffective, whereas verapamil bring out excellent outcomes. Adverse event, such as transient hypotension, was observed in 38.5% of the patients. It resolved rapidly following the drug finished. Therefore, close hemodynamic monitoring is essential during the period of verapamil infusion. In general, verapamil is safe and effective in children and adolescents. Due to the negative inotropic effect of calcium antagonists, verapamil is contraindicated in infants (5). Although a case report had shown that intravenous verapamil in an infant with left fascicular VT was safe and feasible, it is still need be caution in clinical practice (25). In this study, intravenous propafenone was efficacious in three infants with sustained left fascicular VT. Neither intravenous nor oral verapamil was adopted in these three infants. The youngest patient who was prescribed verapamil was 3 years-old in our cohort. The EHRA guideline recommends that the administration of intravenous propafenone to terminate sustained VT in children is safe and effective; however, it is contraindicated in patients with structural heart disease or heart failure (8).

Adenosine-sensitive VT is often seen in outflow tract VT, which is mediated by cAMP-dependent triggered activity and delayed afterdepolarizations. First-line agents for this type of VT include amiodarone, propafenone, and beta-blockers. Intravenous propafenone or amiodarone was effective in two patients with presumed OT-VT in our study, whereas adenosine and lidocaine were ineffective.

Propranolol-sensitive VT is commonly seen in patients without structural heart disease and sometimes manifest as polymorphic VT. The mechanism is related to increased adrenergic-mediated automaticity. Adenosine and overdrive pacing may transiently suppress VT. Beta-blockers are regarded as the first-line agent for this type of VT. We observed this phenomenon in an infant with idiopathic polymorphic VT. This patient presented with frequent non- sustained VT despite the treatment of amiodarone, but was well-controlled following propranolol added.

Long-term oral antiarrhythmic drugs are essential for preventing VT recurrence. In our study, the majority of patients received long-term prophylactic medication. The results indicated an overall favorable prognosis in these patients. Over a mean follow-up of 2.1 years (the longest duration was 7.5 years), VT resolved completely in 41.4% of the patients and was well-controlled in the remaining patients. No deaths or heart failure events were observed during follow-up. Verapamil monotherapy achieved a favorable prophylactic efficacy in the patients with presumed left fascicular VT. Additionally, consistent with a previous study (11), the results suggested an excellent prognosis for infants with idiopathic VT. Nearly two-thirds of these infants achieved complete VT resolution during follow-up. The analyses showed that onset at school age and ventricular rate during VT were significantly associated with VT recurrence during antiarrhythmic treatment. Due to the insufficient sample size in this study, the statistical analyses were underpowered; thus, we have to acknowledge that the results are exploratory with limited statistical robustness.

## 5 Conclusion

In conclusion, idiopathic VT is the predominant type of sustained VT in children and adolescents. In this study, the vast majority of the cases were presumed left fascicular VT. Patients with presumed left fascicular VT exhibit sensitivity to intravenous verapamil, although clinicians must remain vigilant for transient hypotension. Amiodarone or propafenone are viable alternatives for other types of VT. In our cohort, the monotherapy success rate was 73.5% for sustained VT termination. The results of long-term follow-up revealed an overall favorable prognosis. All the patients maintained good cardiac function during follow-up and no sudden cardiac deaths were recorded.

## 6 Limitations

This was a single-center observational study rather than a prospective randomized controlled study; thus, the evidence quality is low. Moreover, due to the small sample size, the statistical power is insufficient. Moreover, we need to observe the long-term prognosis of these patients. In the future, we will conduct a randomized controlled study with a larger sample size to improve the evidence quality.

## Data Availability

The datasets presented in this study can be found in online repositories. The names of the repository/repositories and accession number(s) can be found in the article/Supplementary Material.
